# Innovative approach to support therapeutic proteins’ similarity in hydrodynamic size using high-throughput dynamic light scattering and forced degradation

**DOI:** 10.1038/s41598-025-97377-6

**Published:** 2025-11-22

**Authors:** Ashwinkumar Bhirde, Siri Harish, Nicholas Trunfio, Isabella F. de Luna, William Smith, Qiong Fu

**Affiliations:** 1 Division of Product Quality Research VI, Office of Pharmaceutical Quality Research, Office of Pharmaceutical Quality, Center for Drug Evaluation and Research, U.S. Food and Drug Administration, 10903 New Hampshire Avenue, Silver Spring, MD 20993 USA; 2 Division of Product Quality Research V, Office of Pharmaceutical Quality Research, Office of Pharmaceutical Quality, Center for Drug Evaluation and Research, U.S. Food and Drug Administration, 10903 New Hampshire Avenue, Silver Spring, MD 20993 USA; 3 Division of Product Quality Assessment XIV, Office of Pharmaceutical Quality Assessment III, Office of Pharmaceutical Quality, Center for Drug Evaluation and Research, U.S. Food and Drug Administration, 10903 New Hampshire Avenue, Silver Spring, MD 20993 USA

**Keywords:** Dynamic light scattering, Biosimilars, Principal component analysis, Monoclonal antibody, Insulin, Protein aggregation, Hydrodynamic size, Regularization, Cumulants, Biotechnology, Chemistry, Materials science

## Abstract

Comparative analytical assessment (CAA) between the reference product and the proposed product forms the basis of the biosimilarity demonstration. Though Dynamic Light Scattering (DLS) has been implemented for CAA, its capability beyond signature peak for similarity assessments has remained unexplored. Herein, we have developed a innovative forced degradation based sweet spot method consisting of signature peak, temperature range, increment and hold time using high throughput-DLS (HT-DLS) to show similarity in hydrodynamic size between products. In our study, we used rituximab, its biosimilars, and insulin analogs as model products to demonstrate product similarity in hydrodynamic size (D_h_) size through the HT-DLS sweet spot approach. Our data indicate that temperature range, temperature increment, hold time, and regularization algorithm, all play a role in showing analytical similarity in D_h_ size. Our data also indicate that establishing DLS signature peaks of the products is insufficient to show analytical similarity in D_h_ size distribution. Additionally, the temperature range (sweet spot) varies from product to product. Principal component analysis modeling was used for detailed data interpretation. Overall, our HT-DLS based sweet spot method provided informative data to support similarity in D_h_ size distribution.

**Supplementary Information:**

The online version contains supplementary material available at 10.1038/s41598-025-97377-6.

## Introduction

The development of therapeutic proteins has revolutionized the field of medicine, offering treatments for a variety of diseases that were previously untreatable^1^. Biosimilars, biological products that are highly similar to and have no clinically meaningful differences from an already approved biological product (i.e., the reference product), have revolutionized the pharmaceutical industry. Biosimilars have similar clinical benefits and risks as the reference product, and they may offer a cheaper and more accessible alternative for patients^2^^3^. However, demonstrating biosimilarity is a complex process due to the inherent variability of biological products^4^.

To demonstrate that the proposed product is highly similar to the reference product, the Sponsor must perform comparative analytical assessment (CAA) of the proposed product to the reference product in terms of structure and function. Sponsors utilize various analytical tools to demonstrate similarity in critical quality attributes (CQAs) during comparative assessments. These tools evaluate primary structure, higher-order structure (HOS), post-translational modifications (PTMs) such as glycosylation, and other CQAs that may be affected by changes in the manufacturing process^5^. As part of the demonstration that the proposed product is highly similar to the reference product, applicants conduct comparative forced degradation studies. These forced degradation studies involve subjecting the therapeutic proteins to stress conditions that accelerate degradation. These studies can provide valuable insights into the stability and degradation pathways of the therapeutic proteins^6^. By comparing the degradation pathways of the proposed biosimilar and the reference product, similarity in terms of degradation pathways and rates can be assessed. If the two products have similar degradation pathways, it suggests that they have similar structures and are likely to have similar functions^7^.

Dynamic light scattering (DLS) is a powerful technique primarily used to determine the size distribution profile of small particles in suspension or polymers in solution^8^. DLS has emerged as a critical tool for protein particle characterization, providing valuable insights into protein aggregation, stability, and behavior under various conditions^9^. The primary output of a DLS measurement is a particle size distribution. DLS provides the hydrodynamic radius or commonly used hydrodynamic diameter (D_h_), which is the diameter of a hypothetical hard sphere that diffuses in the same manner as the particle^10^. This information is crucial in understanding the physical properties of proteins in solution^11^. Protein aggregation is a common challenge in the biopharmaceutical industry, as it can affect the efficacy and safety of therapeutic proteins. DLS is an effective tool for detecting and monitoring protein aggregation^12^ and can identify aggregates in a size range from a few nanometers to several micrometers^13^. Traditionally DLS measurements are performed using a cuvette; however, a plate reader-based high throughput system provides better capabilities, like running multiple samples at the same time. High throughput-DLS (HT-DLS) is also used to assess protein stability^14^. By monitoring the size distribution of a protein solution over time or under varying conditions (e.g., temperature, pH, ionic strength), researchers can gain insights into the protein’s stability. Any shift in the D_h_ size distribution can indicate unfolding or aggregation, both of which are signs of instability^15^. While DLS cannot directly measure conformational changes, an increase in the D_h_ size can indicate unfolding or partial unfolding of the protein^16^. Overall, the hydrodynamic size of the particles can be used to monitor changes in the size distribution during forced degradation^17^. Thus by comparing the D_h_ size profiles of the proposed biosimilar and the reference product under stress conditions, we can assess their similarity in terms of stability and aggregation behavior.

**Fig. 1:**
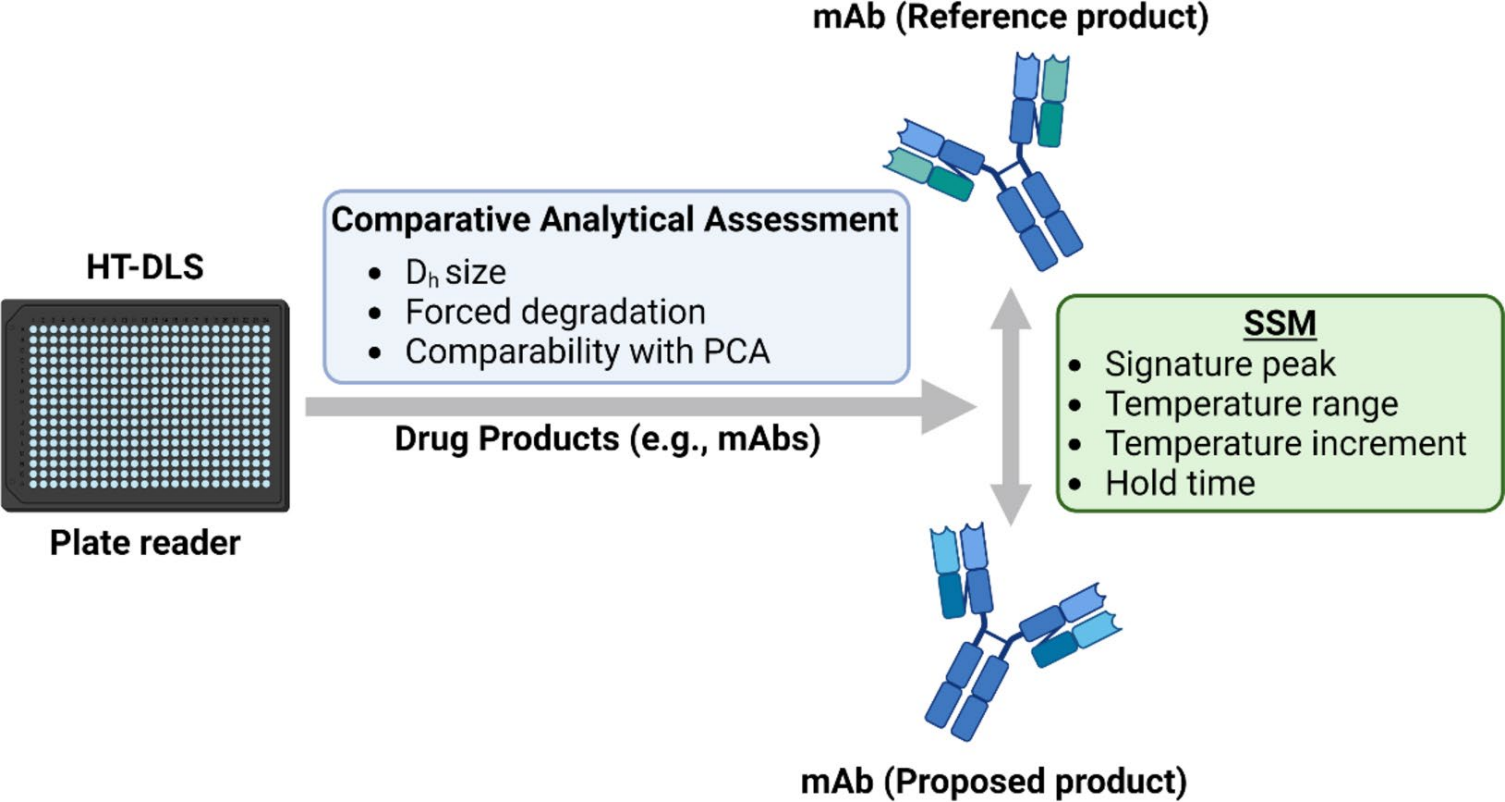
Overview of the high-throughput dynamic light scattering (HT-DLS) Sweet Spot method (SSM) to support analytical similarity of therapeutic proteins like monoclonal antibody (mAb) using dynamic light scattering (D_h_ = hydrodynamic size) and principal component analysis (PCA).

Several previous publications have used DLS as an assay in the comparative analytical assessment^17,18^. However, these studies have been limited to monomer peak in its native state referred here in this manuscript as the “signature peak”. Herein, we have developed an innovative approach of evaluating analytical similarity in D_h_ size distribution through forced thermal degradation using a HT-DLS based sweet spot method (SSM). An overview of the proof-of-concept SSM is provided in Fig. 1. By optimizing a temperature range, temperature increment, and hold time, we were able to compare D_h_ size distribution of approved monoclonal antibody (mAb) biosimilars and their reference products as well as size comparability between insulin products.

## Results

### Developing the sweet spot method for mAb DPs

**Fig. 2:**
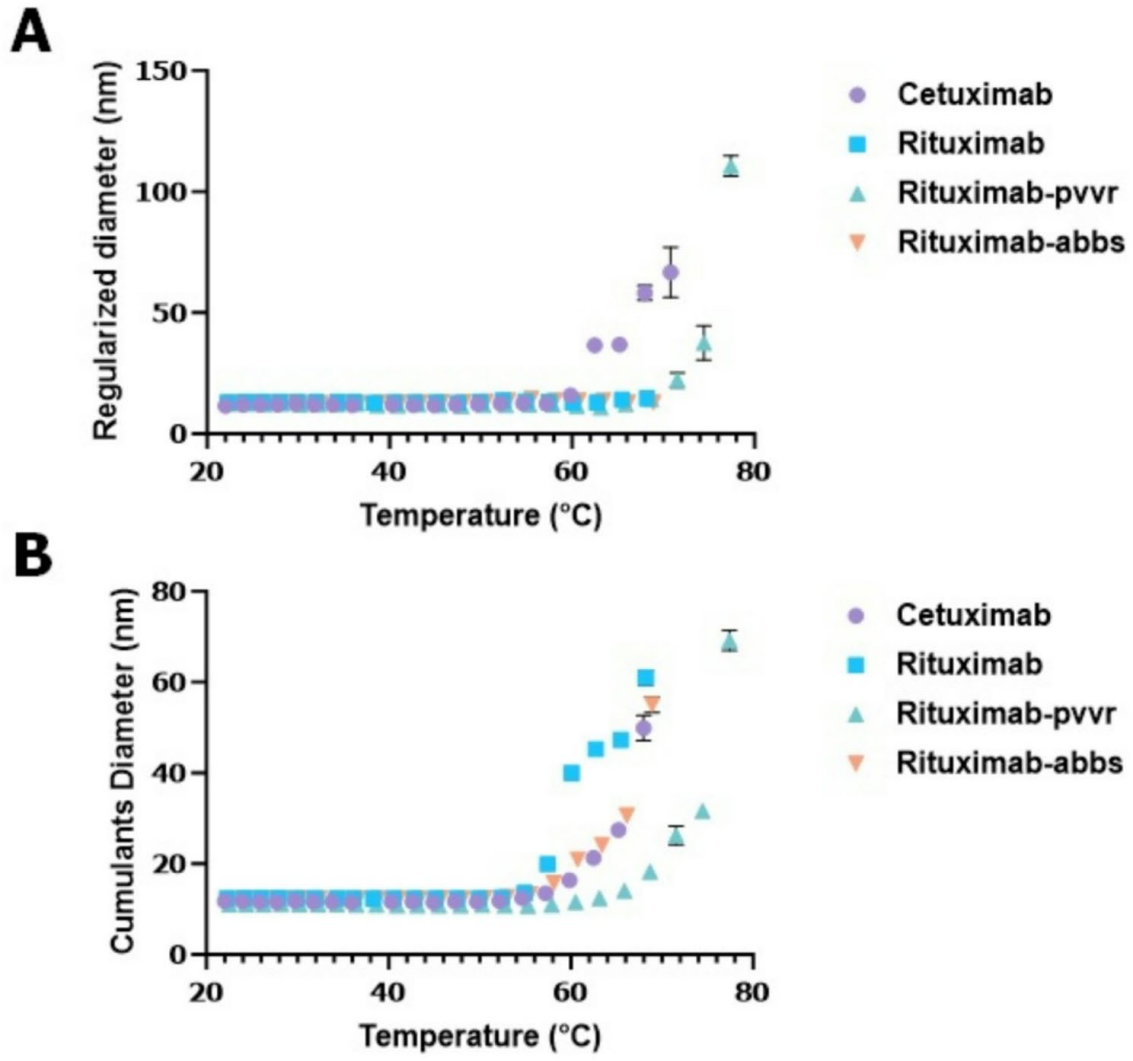
Hydrodynamic (D_h_) size changes of monoclonal antibody (mAb) drug products (DPs) under continuous temperature ramping condition with no hold time and 3℃ increment temperature. (**A**) D_h_ sizes (regularization method) of mAb DPs cetuximab, rituximab and rituximab biosimilars (rituximab-pvvr and rituximab-abbs) stressed between 20 and 80℃ range. (**B**) D_h_ sizes (cumulants method) of mAb DPs cetuximab, rituximab and rituximab biosimilars (rituximab-pvvr and rituximab-abbs) stressed between 20 and 80℃ range. Continuous ramping approach did not provide a clear D_h_ size change pattern between the drugs tested.

To develop the method for comparing size distribution of mAbs, first continuous temperature ramping was tested. For this experiment, rituximab and its biosimilars, rituximab-pvvr and rituximab-abbs, were evaluated alongside cetuximab as a non-biosimilar mAb DP. Cumulants, popularly known as Z-average (measures single species with monomodal D_h_ size distribution), and regularization also known as intensity weighted nonnegative least-squares method, makes no prior assumptions about the shape or form of the D_h_ size distribution in providing information about the aggregates and size distribution are two D_h_ size data processing models used in DLS runs. For all DLS runs conducted throughout the study, both models were used to collect D_h_ data to test their suitability under various conditions tested. Data from the continuous temperature ramping did not provide a clear pattern in D_h_ size change (Fig. 2) for the drug products (DPs) tested.

**Fig. 3:**
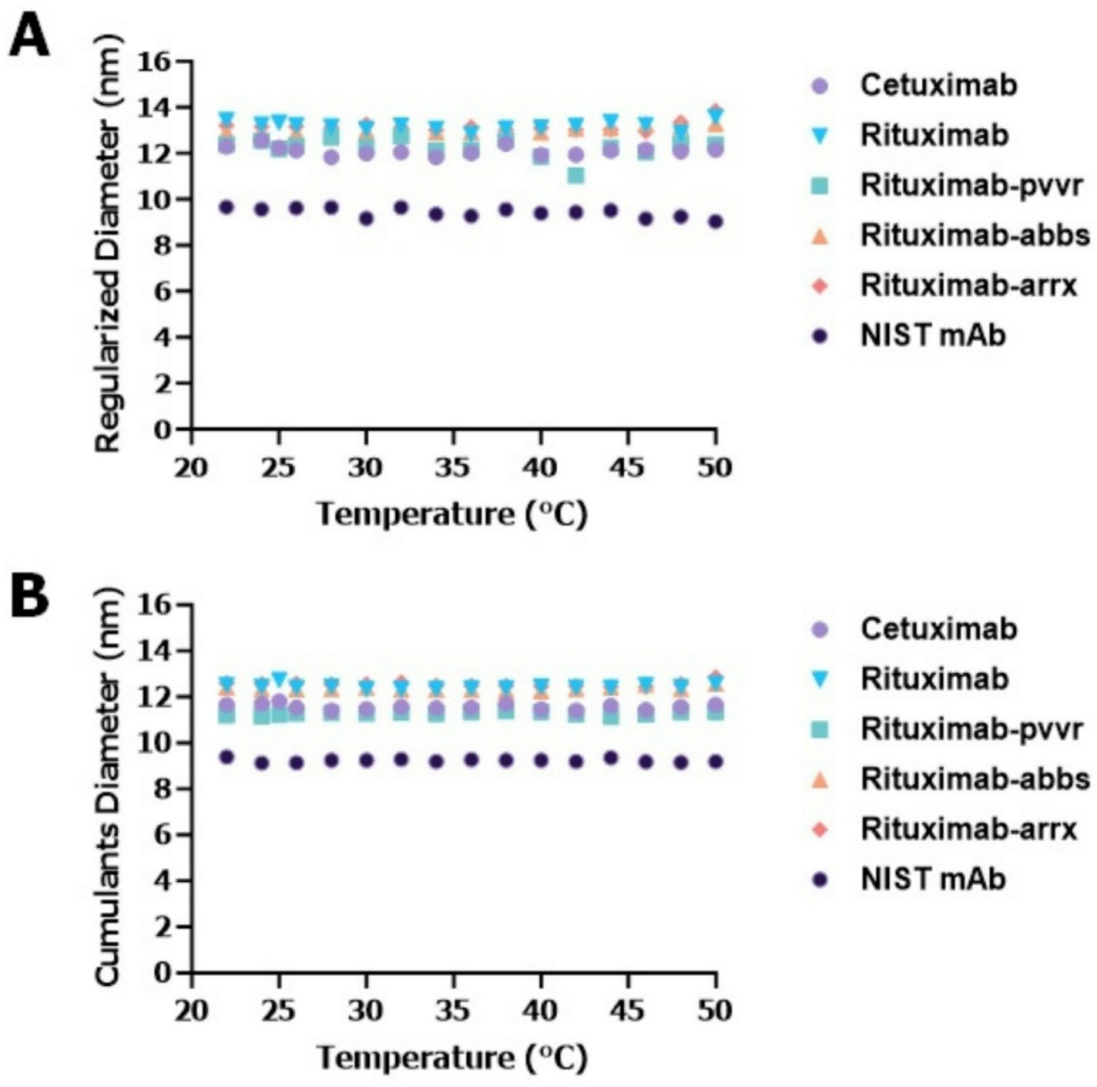
Hydrodynamic (D_h_) size changes of monoclonal antibody (mAb) drug products (DPs) within the no-change temperature ramping condition with 2-minute hold time and 3℃ increment temperature. (**A**) D_h_ sizes (regularization method) of mAb DPs NIST mAb, cetuximab, rituximab and rituximab biosimilars (rituximab-pvvr, rituximab-abbs and riabni) stressed at 22–50℃ range. (**B**) D_h_ sizes (cumulants method) of mAb DPs NIST mAb, cetuximab, rituximab and rituximab biosimilars (rituximab-pvvr, rituximab-abbs and riabni) stressed at 22–50℃ range. Both regularized (**A**) and cumulants (**B**) temperature stress range clearly shows no change in D_h_ size for the controls NIST mAb, cetuximab and between rituximab and its biosimilars.

**Fig. 4:**
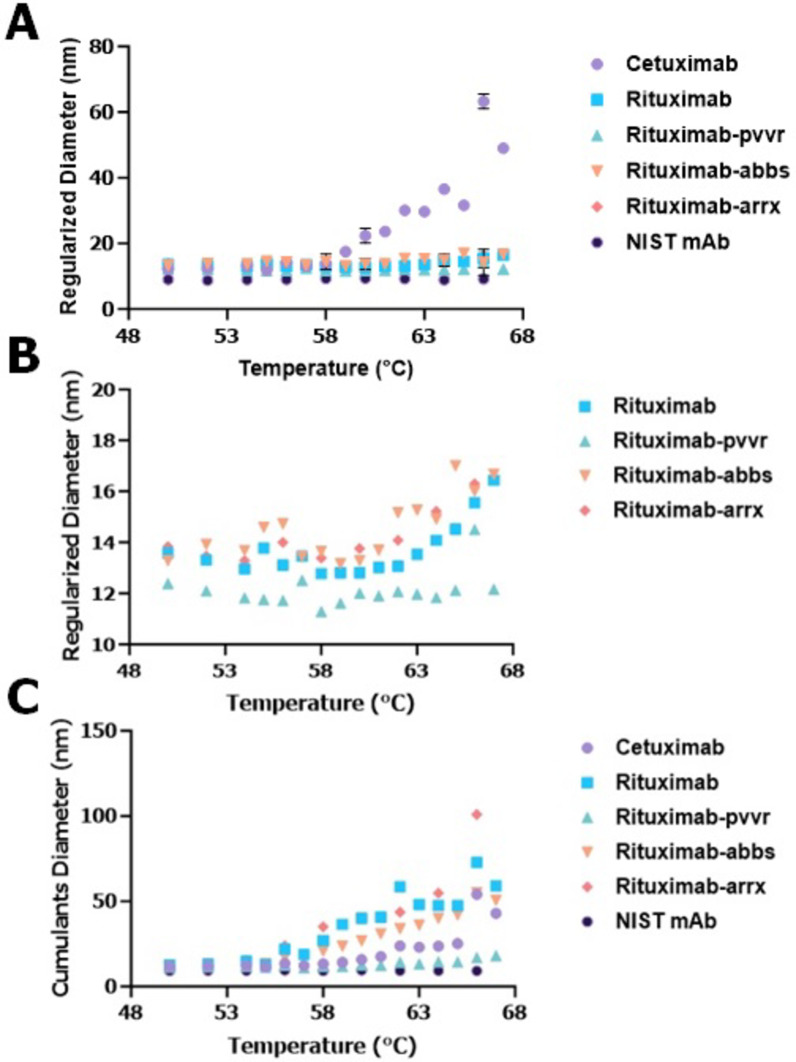
Hydrodynamic (D_h_) size changes of monoclonal antibody (mAb) drug products (DPs) within the Sweet Spot temperature ramping condition with 2-minute hold time and 3℃ increment temperature. (**A**) D_h_ sizes (regularization method) of mAb DPs NIST mAb, cetuximab, rituximab and rituximab biosimilars (rituximab-pvvr, rituximab-abbs and rituximab-arrx) stressed at 50–67℃ range. (**B**) D_h_ sizes (regularization method) of mAb DPs rituximab and rituximab biosimilars (rituximab-pvvr, rituximab-abbs and rituximab-arrx) stressed at 50–67℃ range. (**C**) D_h_ sizes (cumulants method) of mAb DPs NIST mAb, cetuximab, rituximab and rituximab biosimilars (rituximab-pvvr, rituximab-abbs and rituximab-arrx) stressed at 50–67℃ range. Regularized (**A**,**B**) sweet spot range clearly shows D_h_ size similarity pattern between rituximab and its biosimilars.

Next, we tested the mAbs under temperature ramping with a set hold time, which provided adequate duration to observe meaningful differences or similarities between two products. For this test, rituximab (rituximab, rituximab-pvvr, rituximab-abbs, and rituximab-arrx) and cetuximab products were evaluated along with NIST mAb, which was used as a well-characterized reference standard, to differentiate drug products, and to demonstrate the suitability of the assay. Data from this experiment indicated that the DPs tested did not show any change in their D_h_ size up to 50℃ (Fig. 3). After determining the temperature range in which D_h_ size did not change, the same DPs were then tested at a higher temperature range. For this step, the DPs were evaluated between 50 and 70℃ with a 2-minute hold time and 3℃ increment temperature (Fig. 4) using the event schedule from the Dynamics software. All the DPs were tested at their original concentration. Results observed from this experiment clearly indicated that these biosimilars and their reference product had a similar D_h_ size changing pattern compared to the other DPs tested. Initially, optimization with the sweet spot method was performed using rituximab and only one of its biosimilars to minimize costs. Once the similarity pattern was established using the sweet spot method, then more drugs were added to ensure the method worked. The validity of the approach was confirmed with rituximab and 3 biosimilars showing similar size change patterns. In short, the optimized hold time, temperature range, and increment formed the core of the sweet spot method.

### CAA of mAb DPs using PCA

**Fig. 5:**
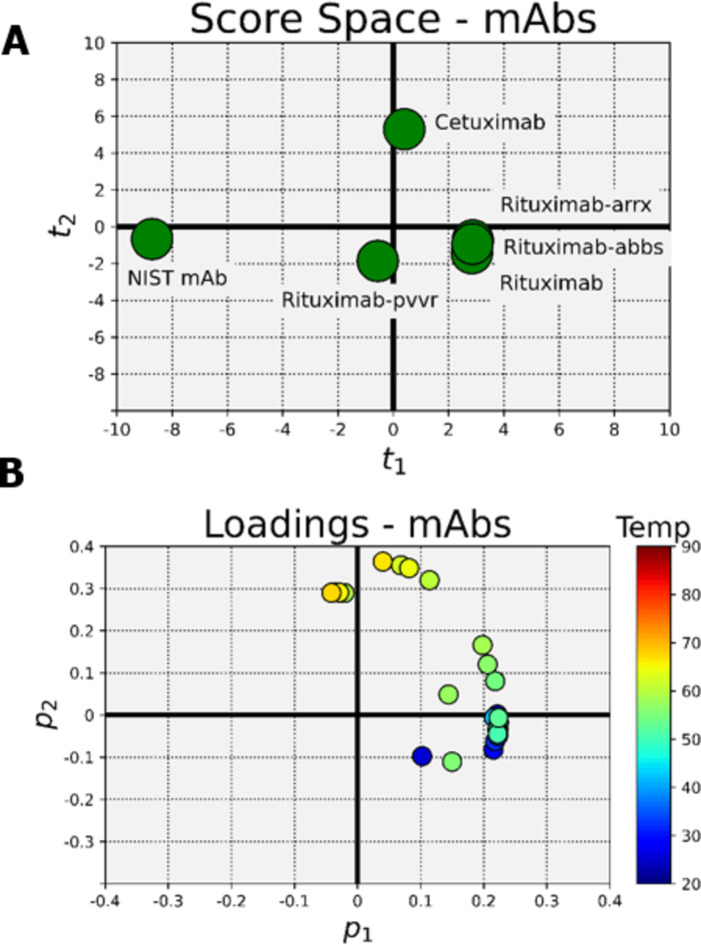
Principal component analysis modeling of the Hydrodynamic (D_h_) size changes of monoclonal antibody (mAb) drug products (DPs) within the Sweet Spot temperature ramping condition with 2-minute hold time and 3℃ increment temperature. (**A**) The score space and (**B**) loadings for the mAb products. They show that rituximab and its biosimilars have similar D_h_ size change pattern across the entire temperature range.

To elucidate the variation between mAbs at different temperatures, we implemented principal component analysis (PCA) modeling (refer to PCA modeling in “Materials and Methods”). The score space for the mAbs (Fig. 5A) showed that the first principal component (x-axis) primarily captured the variability distinguishing NIST mAb from the other mAbs. The loadings in Fig. 5B showed that the first principal component may be interpreted as being positively correlated with the low temperature measurements. Taken together, this indicated that NIST mAb having a smaller diameter than the other mAbs at low temperatures may be responsible for the pattern seen in the score space. The score space showed that the second principal component (y-axis) primarily captured the variability which distinguished cetuximab from the other mAbs. The loadings showed that the second principal component may be interpreted as being positively correlated with the high temperature measurements. Taken together these data indicated that cetuximab diameter increasing more rapidly than the others as the temperature was increased may be responsible for the pattern seen in the score space. The grouping of rituximab, rituximab-pvvr, rituximab-arrx and rituximab-abbs on both principal components indicated that they had similar D_h_ sizes across the entire temperature range.

### Sweet spot method under diluted DP concentration

**Fig. 6:**
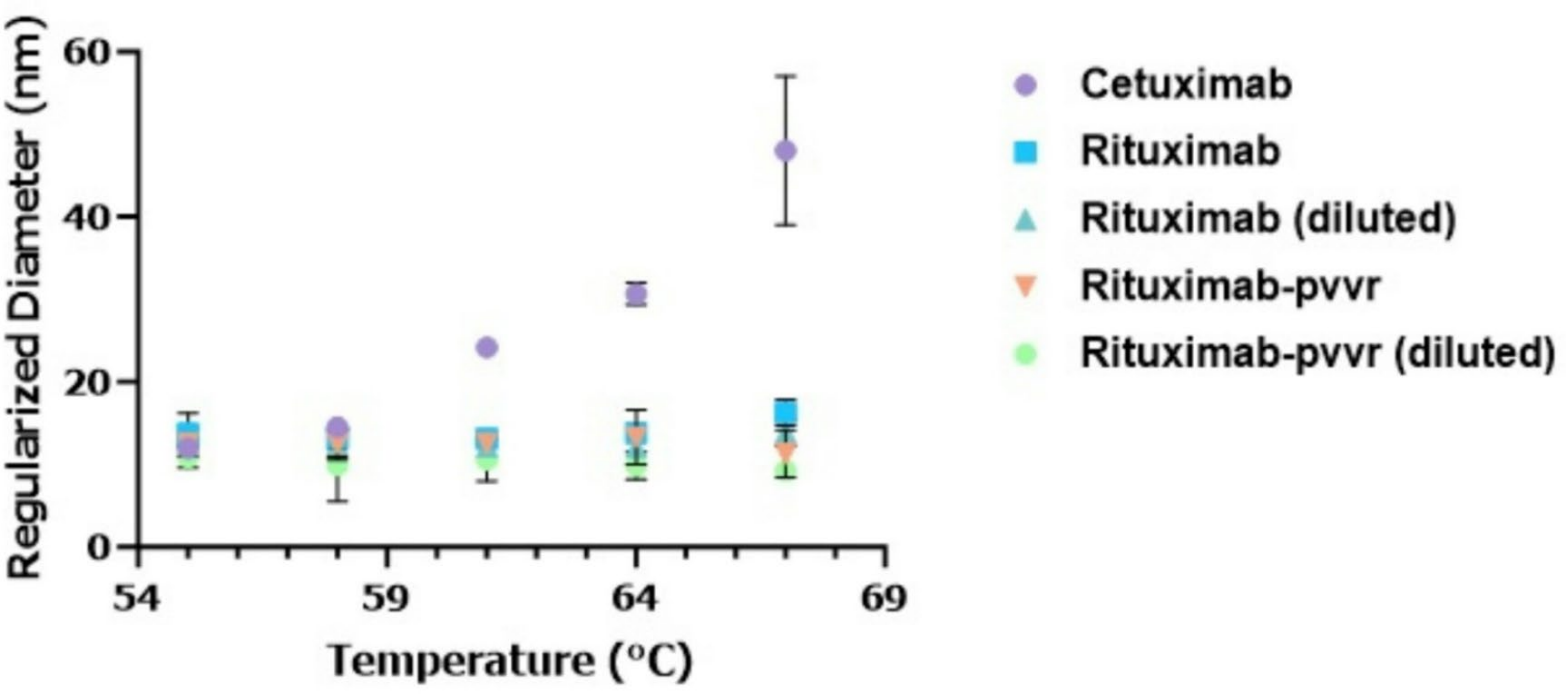
Hydrodynamic (D_h_) size changes of diluted (2 mg/mL) and undiluted (10 mg/mL) monoclonal antibody (mAb) drug products (DPs) within the Sweet Spot temperature ramping condition with 2-minute hold time and 3℃ increment temperature. D_h_ sizes (regularization method) of diluted and undiluted rituximab and rituximab biosimilar rituximab-pvvr, and cetuximab (undiluted 2 mg/mL) stressed at 55–67℃ range. Regularized sweet spot range clearly shows D_h_ size similarity between rituximab and its biosimilar rituximab-pvvr even when diluted.

**Fig. 7:**
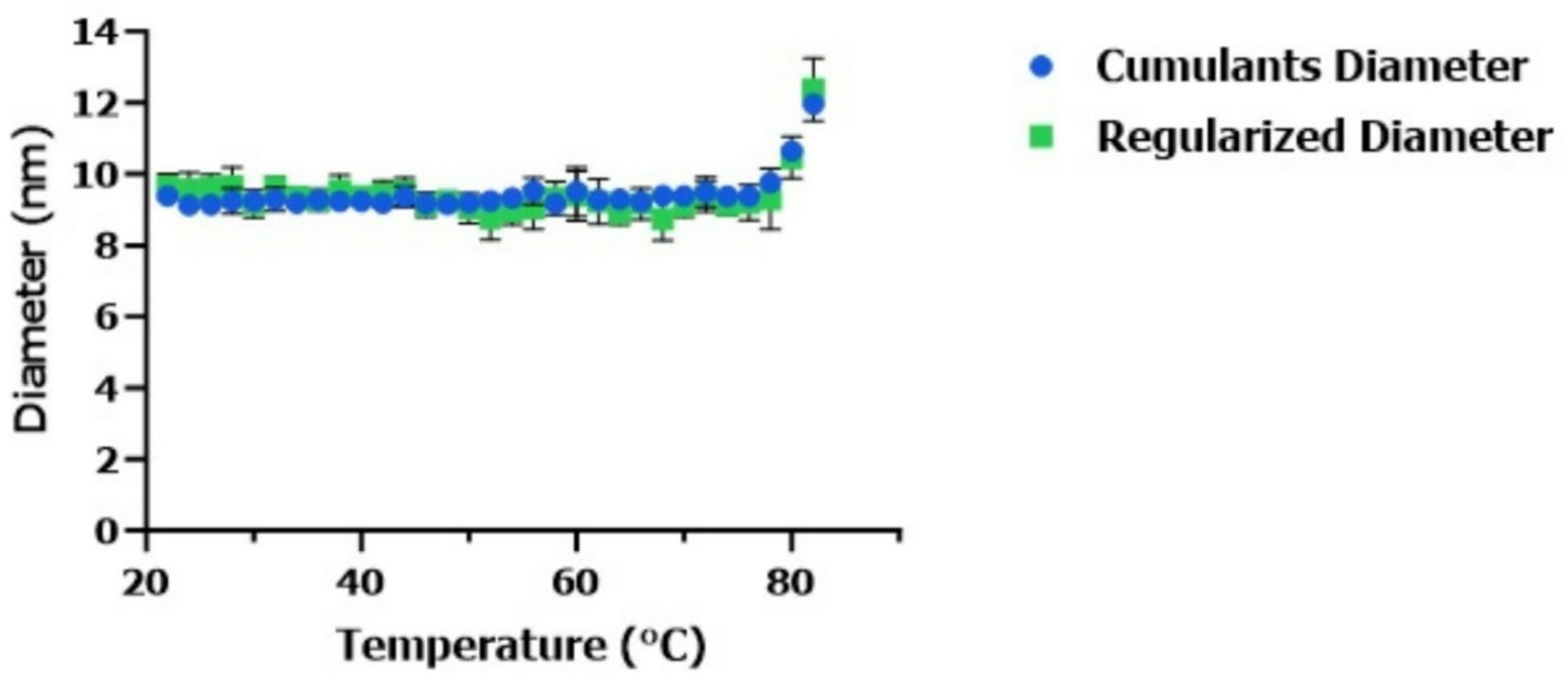
Hydrodynamic (D_h_) size changes of NIST mAb covering the no change temperature range and the mAb sweet spot temperature range with 2-minute hold time and 3℃ increment temperature. D_h_ sizes (cumulants (blue) and regularization (green) method) of NIST mAb stressed at 22–82℃ range. Both cumulants and regularized data processing algorithms shows no change in D_h_ size for the mAb reference standard control NIST mAb for the temperature range between 22–79℃.

To understand the effectiveness of our approach for lower concentration DPs, we tested the mAb DPs under diluted conditions. For this test, cetuximab (2 mg/mL original concentration), rituximab (diluted (2 mg/mL) and undiluted (10 mg/mL)) and rituximab biosimilar rituximab-pvvr (diluted (2 mg/mL) and undiluted (10 mg/mL)) were evaluated. Rituximab and its biosimilar were diluted to similar concentration as cetuximab. Data (Fig. 6) obtained indicated diluted rituximab-pvvr and the reference product rituximab had a similar D_h_ size change pattern compared to undiluted products, suggesting the D_h_ size change pattern is not impacted by protein concentration. To establish a control standard next, we tested NIST mAb as a suitable control reference standard that can be implemented during method development for testing mAb DPs. For this test, we evaluated NIST mAb under the hold time and increment temperature ramping approach from 22 to 82℃ (Fig. 7). Data showed there was no change in the NIST mAb D_h_ size up to 79℃.

### Developing the sweet spot method and PCA for fast-acting insulin DPs

**Fig. 8:**
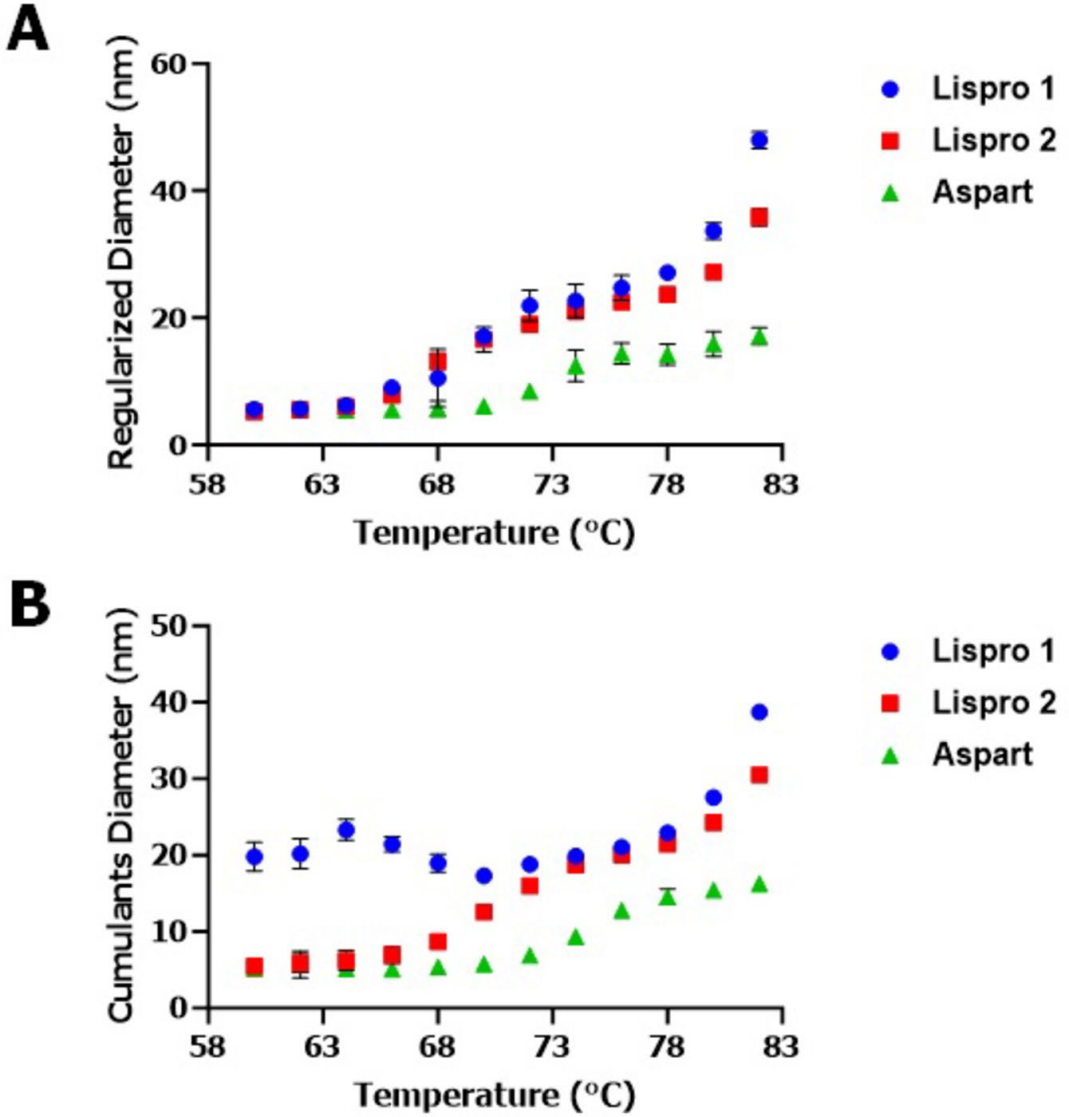
Hydrodynamic (D_h_) size changes of insulin drug products (DPs) within the Sweet Spot temperature ramping condition with 2-minute hold time and 3℃ increment temperature. (**A**) D_h_ sizes (regularization method) of insulin DPs aspart, lispro 1 and lispro 2 stressed at 60–80℃ range. (**B**) D_h_ sizes (cumulants method) of insulin DPs aspart, lispro 1 and lispro 2 stressed at 60–80℃ range. Regularized (**A**) sweet spot range clearly shows D_h_ size similarity between the insulin lispro DPs lispro 1 and lispro 2 which have similar formulation.

Once we established the method for mAbs, we compared insulin lispro, insulin aspart, and glulisine DPs with the sweet spot method. For this approach, we evaluated the insulin DPs from 55 to 80℃ (Fig. 8 and Figure S1). Results observed from this experiment clearly indicated that insulin lispro DPs had a similar D_h_ size change pattern compared to the other insulin DPs tested. Next, PCA modeling was used to facilitate a deeper understanding of the fast-acting insulin DP differences with increasing temperature. The score space for the insulin products (Figure S2A) showed that the first principal component (x-axis) primarily captured the variability distinguishing insulin lispro (admelog) and aspart (novolog) and insulin lispro (humalog) and insulin glulisine (apidra). The second principal component (y-axis) captured the variability distinguishing between insulin lispros (humalog and admelog) from the other insulin DPs. The loadings in Figure S2B showed that the first principal component was positively correlated with the low and high temperature measurements. The loadings showed that the second principal component was positively correlated with higher temperature. Taken together, this indicated that the diameter of insulin lispro DPs (humalog and admelog) increasing more rapidly as the temperature increased relative to the other two DPs may be responsible for the pattern seen in the score space. However, as each of the four DPs projected into different quadrants, it is not possible to conclude if this is a real trend or if the analysis is only saying that the DPs are different using PCA modeling. The continuous temperature ramping approach was not successful for insulin DPs as well (Figure S3).

### Establishing signature peaks for mAb and insulin DPs

**Fig. 9:**
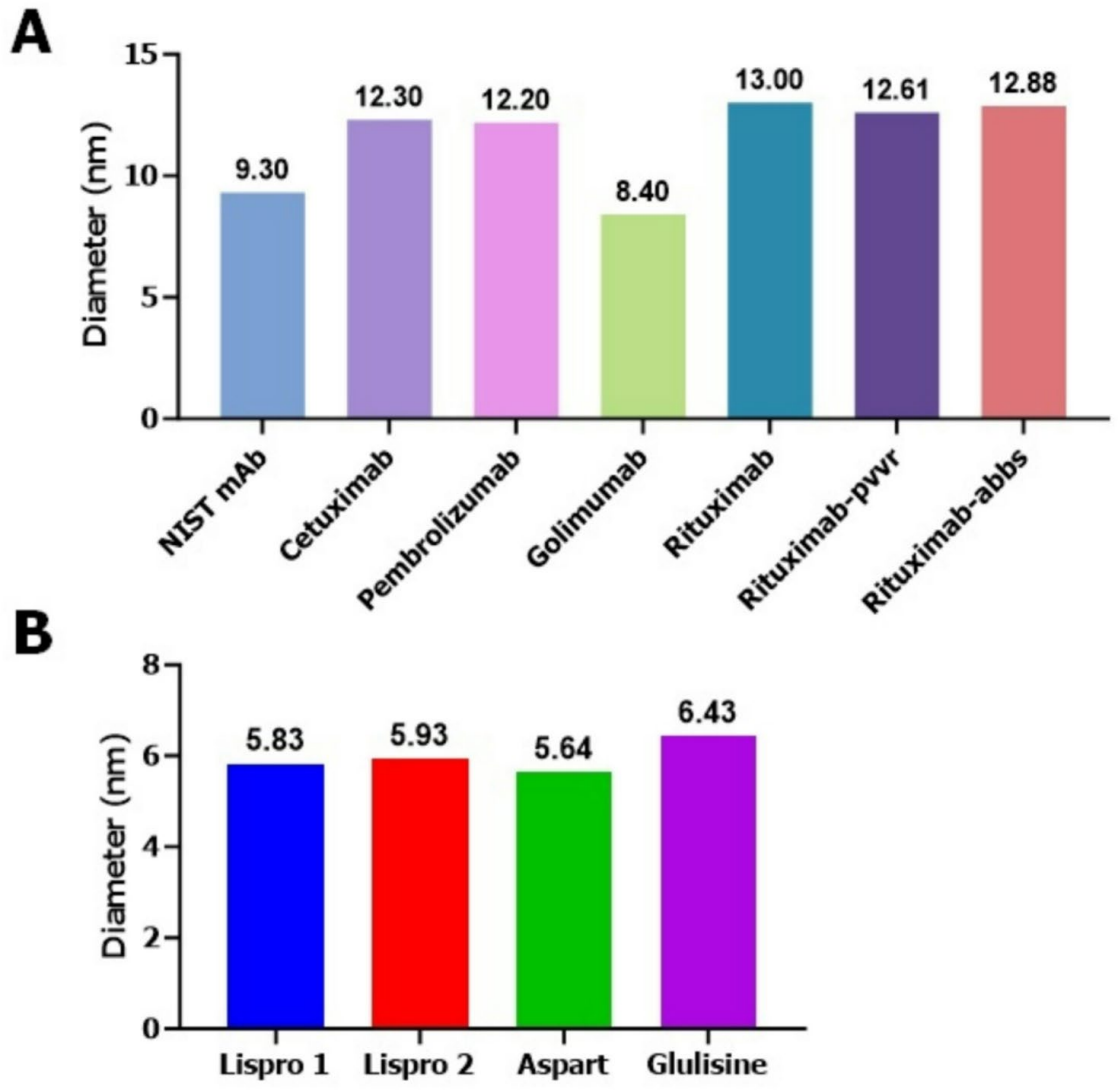
Signature hydrodynamic (D_h_) size peaks of mAb and insulin DPs in solution at room temperature. (**A**) mAb DPs NIST mAb, cetuximab, pembrolizumab, golimumab, rituximab, rituximab-pvvr and rituximab-abbs were tested for D_h_ size at 22℃. (**B**) insulin DPs lispro 1, lispro 2, aspart and glulisine were tested for D_h_ size at 22℃. All mAb DPs show a signature peak between 8 and 13 nm (**A**) and all insulin DPs tested show a signature peak between 5–6 nm (**B**).

We wanted to test if all mAb and insulin DPs have different or similar D_h_ sizes at room temperature. For this test we evaluated a total of eight mAb DPs including NIST mAb, cetuximab, pembrolizumab, golimumab, rituximab, its biosimilars rituximab-pvvr and rituximab-abbs, as well as four insulin DPs (insulin lispros, insulin aspart, and insulin glulisine) in solution at 22℃ to establish their signature peaks. Data obtained (Fig. 9 and Figures S4, S5) indicated all the mAbs tested showed a signature D_h_ size peak between 8 and 13 nm while all the fast-acting insulin DPs tested showed a signature D_h_ size peak between 5 and 6 nm. To pick the appropriate data processing algorithms for mAb DPs, data from both cumulants and regularization data processing algorithms were evaluated. Our data (Figure S6) indicated that for our customized algorithm-based HT-DLS sweet spot method, regularization was the most suitable data processing algorithm.

## Discussion

Herein, we report the development of an innovative DLS-based method (Fig. 1) which can be leveraged to assess analytical similarity in D_h_ size distribution based on hydrodynamic size peaks of mAb drugs products and insulin analogs. Both continuous and discrete temperature ramping were tested to evaluate D_h_ size pattern similarity between mAb biosimilar DPs and their reference DP as well as between insulin DPs.

First, we tested continuous temperature ramping for mAb DPs at their original concentrations, however no set pattern of D_h_ size changes could be observed (Fig. 2). Next, we tested thermal ramping with specific hold times and temperature increments using customizable event schedule algorithms provided in the Dynamics software. Initially, we tested the mAbs for D_h_ size change for up to 50℃. No changes in D_h_ size were observed for any mAb DPs tested, including the biosimilars (Fig. 3). We then evaluated mAb DPs for D_h_ size change at temperatures > 50℃ using the same customized event schedule. These experiments revealed a pattern of D_h_ size changes, with the biosimilars and the reference product displaying similar D_h_ size change patterns while other DPs tested showed different D_h_ size change patterns (Fig. 4). After optimizing the temperature range, increment, and sample hold time in the temperature range of 50–67℃ with a 2-minute hold time and 3℃ increment temperature, the temperature range of 50–67℃ was considered a sweet spot range to show similar D_h_ size distributions for the mAb DP biosimilars tested. Several studies have used principal component analysis (PCA)^19^ modeling for better data interpretation^20^. PCA modeling of the mAb DPs (Fig. 5) indicated the closeness of the biosimilars with the reference DP when compared to the two controls (cetuximab and NIST mAb). The grouping of rituximab, rituximab-pvvr, rituximab-arrx, and rituximab-abbs on both principal components indicated that they are similar across the entire temperature range.

To determine if the DPs tested would show D_h_ size similarity at lower concentrations, we diluted the DPs and tested under the sweet spot range (Fig. 6). Experiments with the diluted, undiluted biosimilar, and reference DPs showed similar results and showed that our innovative method can also be applied to low concentration DPs. Next, we tested NIST mAb to evaluate its proposed use as a standard reference control during comparative analytical assessment of mAb DPs. The same customized event schedule algorithm was used to test the NIST mAb over the entire temperature range from 22 to 80℃. Our experimental data clearly showed that the NIST mAb D_h_ size change pattern was different from the rest of the mAb DPs tested (Fig. 7) and can be used as a control during method development and establishment.

Once we established the method for mAbs, we then wanted to evaluate the new method for non-mAb DPs. For these experiments, we chose insulin DPs. The sweet spot method that was developed for mAbs was implemented here. Unlike mAb DPs, the fast-acting insulin DPs did not show D_h_ size change up to 60℃ (Fig. 8). Our experimental data indicated the sweet spot range for insulin DP was from 60 to 80℃ (Fig. 8). The data observed from insulin experiments also indicated that the sweet spot range varied for DP types and needed to be established separately for each insulin product. PCA modeling data (Figure S2) showed the closeness of the insulin DPs and taken together, this indicated that Lispro 2 and Lispro 1 diameters increased more rapidly as the temperature increased relative to the other two DPs. Similar to mAb DPs, continuous temperature ramping did not provide any useful D_h_ size change pattern for insulin DPs (Figure S3). Past publications have used DLS as a tool to show analytical similarity. However, these studies were limited to the DP monomer peak D_h_ size alone in its native state and were also sometimes based on the incorrect assumption of the DP monomer signature peak^16,18^. Therefore, within this study a method was developed to compare the similarity in D_h_ size pattern under thermal stress conditions for the first time.

Next, we wanted to test the signature D_h_ size peaks for the mAb biosimilars and insulins to find out if these DPs have unique sizes compared to DPs from other drug classes. Experimental data (Fig. 9 and Figures S4-S5) from these runs clearly indicated D_h_ size signature peaks for these mAb DPs tested ranged between 8 and 13 nm, while for insulin DPs tested the D_h_ size signature peaks ranged from 5 to 6 nm. The results from this experiment also indicated that evaluating signature peaks alone is insufficient in establishing analytical similarity using D_h_ size alone since the mAbs and insulin DPs tested each had the same signature peak ranges regardless of being biosimilar or not. The cumulants data processing algorithm assumes each sample as monodispersed and provides the average D_h_ size data of the sample, while regularization algorithm can resolve multiple peaks and provides data for all species (particle sizes or polydisperse samples) identified during the DLS experimental run. Cumulants (Z-average) size data is applicable for the no change zones (Figs. 2 and 5) and control (i.e., signature peak, Fig. 7) samples which is the no D_h_ size change zone. Cumulants data fit is known to be good for only monomodal monodisperse^10^ or polydisperse samples, whereas regularization fit is good for both monomodal and multimodal monodisperse or polydisperse samples. Our experimental data from both mAb and insulin DPs indicated data obtained from the regularization data processing algorithm (Figure S6) should be used when implementing our method for D_h_ size similarity. Cumulants and regularization data were similar within the no change zone (Figure S6A), however cumulants z-average D_h_ at higher temperatures did not provide size change pattern. Overall, our study data indicated measuring D_h_ size using HT-DLS under thermal stress within the sweet spot temperature range (Table S1) can be a simple, straightforward, and useful approach for showing analytical similarity in D_h_ size distribution of mAb and insulin DPs in solution format. This study provided information on efficient D_h_ size pattern change detection using HT-DLS analytical technology and provided information on best practices for assessing and reporting size-related quality attributes when submitting analytical similarity data to the Agency.

## Conclusion

Here in our study, we describe an innovative approach that can assess similarity in the hydrodynamic size pattern under forced degradation of therapeutic protein DPs by HT-DLS. Our data showed that a mAb biosimilar and its reference product have similar hydrodynamic size change pattern within a sweet spot high-temperature range. The method was optimized by finding the best sample hold time, temperature increment, and range along with signature peak for hydrodynamic size similarity. PCA modeling can be used to facilitate interpretation of the hydrodynamic size pattern towards hydrodynamic size similarity assessment. Our data demonstrated that cumulant data processing and continuous ramping did not help in method development. Data indicates signature peak alone is insufficient in proving hydrodynamic size similarity. Our method is currently applicable for DPs in solution only. The sweet spot approach for hydrodynamic size similarity will be a valuable addition to the existing methods for biosimilarity assessment.

## Materials and methods

### Monoclonal antibodies (mAbs)

NIST mAb (RM 8671) was provided as one internal-threaded polypropylene cryovial containing 800 µL of 10 mg/mL NIST IgG1κ monoclonal antibody (NISTmAb) in 12.5 mmol/L L-histidine, 12.5 mmol/L L-histidine HCl (pH 6.0).

Cetuximab (Erbitux, 2 mg/mL) [ Single-dose, 50-mL vial; 100 mg Cetuximab; each mL contains 0.31 mg citric acid monohydrate, 1.1 mg glycine, 0.015 mg polysorbate 80, 8.47 mg sodium chloride, 2.3 mg sodium phosphate dibasic heptahydrate, 0.2 mg sodium phosphate monobasic monohydrate, and water for injection, USP.].

Rituximab (Rituxan, 10 mg/mL), [Single-dose vial; each mL of solution contains 0.7 mg of polysorbate 80, 9 mg of sodium chloride, 7.35 mg of sodium citrate, and water for injection, USP. pH is 6.5.].

Rituximab-pvvr (Ruxience, 10 mg/mL), [Single-dose 100 mg vial; each mL of solution contains 0.056 mg of edetate disodium dihydrate, 1.2 mg of L-histidine, 2.57 mg of L-histidine hydrochloride monohydrate, 0.2 mg of polysorbate 80, 85 mg of sucrose, and water for injection, USP.].

Rituximab-abbs (Truxima, 10 mg/mL) [Single-dose 100 mg vial; each mL of solution contains 0.7 mg of polysorbate 80, 9 mg of sodium chloride, 7.35 mg of tri-sodium citrate dihydrate, and water for injection, USP.].

Rituximab-arrx (Riabni, 100 mg per 10 mL, 10 mg/mL) Each mL of solution contains 10 mg rituximab-arrx, polysorbate 80 (0.7 mg), sodium chloride (9 mg), sodium citrate dihydrate (7.35 mg), and Water for Injection, USP. Hydrochloric acid is used to adjust the buffer solution pH. The pH is 6.5.

Pembrolizumab (Keytruda) injection is a sterile, preservative-free, clear to slightly opalescent, colorless to slightly yellow solution for intravenous use. Each vial contains 100 mg of pembrolizumab in 4 mL of solution. Each 1 mL of solution contains 25 mg of pembrolizumab and is formulated in: 1.55 mg L-histidine, 0.2 mg polysorbate 80, 70 mg sucrose, and Water for Injection, USP.

Golimumab (Simponi Aria) 50 mg of Simponi per 0.5 mL of solution, 1.14 mg L-histidine, 6.42 mg L-histidine monohydrochloride monohydrate, 180 mg sorbitol, 0.6 mg polysorbate 80, and water for injection. Simponi does not contain preservatives.

### Insulin DPs

Lispro 1 (Humalog injection), 10 mL multiple-dose vial; each mL contains 100 units of insulin lispro, 16 mg of glycerin, 1.88 mg of dibasic sodium phosphate, 3.15 mg of metacresol, zinc oxide content adjusted to prove 0.0917 mg zinc ion, trace amounts of phenol, and water for injection. Hydrochloric acid 10% and/or sodium hydroxide 10% may be added to adjust pH.

Lispro 2 (Admelog injection), One 3 mL vial; each mL contains 100 units of insulin lispro, 16 mg of glycerin, 1.88 mg of dibasic sodium phosphate, 3.15 mg of metacresol, zinc oxide content adjusted to prove 0.0917 mg zinc ion, and water for injection. The pH is adjusted by addition of aqueous solutions of hydrochloric acid and/or sodium hydroxide.

Aspart (Novolog injection), 10 mL multiple-dose vial; each mL contains 100 units of insulin aspart, and inactive ingredients: 1.25 mg of disodium hydrogen phosphate, 16 mg of glycerin, 1.72 mg of metacresol, 1.5 mg of phenol, 0.58 mg of sodium chloride, 19.6 mcg of zinc, and water for injection, USP. Hydrochloric acid 10% and/or sodium hydroxide 10% may be added to adjust pH.

Glulisine (Apidra injection), 10 mL multiple-dose vial each mL contains 100 units (3.49 mg) of insulin glulisine, 3.15 mg of metacresol, 6 mg of tromethamine, 5 mg of sodium chloride, 0.01 mg of polysorbate 20, and water for injection. The pH is adjusted by addition of aqueous solutions of hydrochloric acid and/or sodium hydroxide.

### Experiment design

30 µL of drug was pipetted into eight wells each. The plate was centrifuged at 4700 RPM for 3 min to remove any air bubbles, and bottom of the plate was wiped with lens cleaning paper to ensure it was clean. The plate was then placed into the DynaPro plate reader, and a fixed temperature control experiment was run at 22 °C with 5 acquisitions and a 3-second acquisition time. To prevent sample evaporation under thermal stress 10 µL of paraffin oil were pipetted into each well, followed by centrifugation at 4700 RPM for 3 min. For simultaneous collection and calculation of hydrodynamic diameter (D_h_) size data, the buffer (DP formulation) viscosity was assumed to be unity (water viscosity) for all drug measurements, and the laser wavelength was set to 826 nm. D_h_ size data was based on the Rayleigh spheres model. A continuous temperature ramp experiment (Fig. 2) was conducted, collecting 10 acquisitions with a 2-second acquisition time. For the sweet spot method, the temperature started at 22 °C and ended at 82 °C, with a 2 °C increment every scan and a 10-minute hold time. Customized unique event schedule algorithms were used to run the specific experiments.

D_h_ size of the DPs tested were obtained by the Dynamics software’s built-in algorithms for processing cumulants and regularization data. Temperature ramp event schedule^10^ provided in the Dynamics software was used to develop the customized sweet spot method. Details of the algorithms are restricted to the vendor of the software. The D_h_ sizes (*n* = 3) were further evaluated for standard error.

### Principal component analysis (PCA) modeling

Principal Component Analysis (PCA) is a tool used for dimensionality reduction in data analysis. It reduces a set of correlated features, **X**, such as particle sizes at different temperatures, into a smaller number of principal components (PCs) that better capture the variance in the raw data. The first principal component captures the most variance present in the features with each of the A subsequent PCs capturing progressively smaller amounts of the variance according to equation *XYZ*. The loadings, **p**, represent the orientation of the PCs to the original features, and they are selected to maximize the variance of the scores, **t**, which are the value of each observation on the new PCs. In practice, PCA can uncover underlying patterns in the raw data and highlight relationships between observations.

$$X=\mathop \sum \limits_{{i=1}}^{A} {t_i}p_{i}^{T}+E\left( {*XYZ*} \right)$$

PCA was performed in SIMCA 18 (Sartorius) where the number of principal components to extract was selected by maximizing the portion of variability in the raw data captured by the principal components. The D_h_ size values at various temperatures were used as the set of features, X. The results were plotted using Python.

### Disclaimer

This manuscript reflects the views of the authors and should not be construed to represent the Food and Drug Administration’s views or policies. Certain commercial equipment, instruments, or materials are identified in this presentation to foster understanding. Such identification does not imply recommendation or endorsement by FDA, nor does it imply that the materials or equipment identified are necessarily the best available for the purpose.

## Electronic supplementary material

Below is the link to the electronic supplementary material.

Supplementary Material 1

## Data Availability

The datasets generated during and/or analyzed during the current study are available from the corresponding author on reasonable request.

## References

[1] Joshi, D. et al. Biosimilars in oncology: latest trends and regulatory status. *Pharmaceutics* **14** (12). (2022). 10.3390/pharmaceutics14122721 PMC9784530 36559215

[2] May, M. B., Taucher, K. D. & Vogel, W. H. Practical considerations for integrating biosimilars into clinical practice. *J. Adv. Pract. Oncol.* **12** (4), 431–438. 10.6004/jadpro.2021.12.4.7 (2021). 34123479 10.6004/jadpro.2021.12.4.7 PMC8163250

[3] Bachu, R. D. et al. Oncology biosimilars: new developments and future directions. *Cancer Rep. (Hoboken)*. **5** (11), e1720. 10.1002/cnr2.1720 (2022). 36195576 10.1002/cnr2.1720 PMC9675387

[4] Nupur, N., Joshi, S., Gulliarme, D. & Rathore, A. S. Analytical similarity assessment of biosimilars: global regulatory landscape, recent studies and major advancements in orthogonal platforms. *Front. Bioeng. Biotechnol.* **10**, 832059. 10.3389/fbioe.2022.832059 (2022). 35223794 10.3389/fbioe.2022.832059 PMC8865741

[5] Joshi, S. & Rathore, A. S. Assessment of Structural and Functional Comparability of Biosimilar Products: Trastuzumab as a Case Study. *BioDrugs.*** 34**(2), 209–223. 10.1007/s40259-020-00404-3. (2020). 10.1007/s40259-020-00404-3 31975160

[6] Dyck, Y. F. K. et al. Forced degradation testing as complementary tool for biosimilarity assessment. *Bioeng. (Basel)*. **6** (3). 10.3390/bioengineering6030062 (2019). 10.3390/bioengineering6030062 PMC6783961 31330921

[7] Pisupati, K. et al. Biosimilarity under stress: A forced degradation study of Remicade(R) and Remsima. *MAbs* **9** (7), 1197–1209. 10.1080/19420862.2017.1347741 (2017). 28787231 10.1080/19420862.2017.1347741 PMC5627586

[8] Bhirde, A. A., Hassan, S. A., Harr, E. & Chen, X. Role of albumin in the formation and stabilization of nanoparticle aggregates in serum studied by continuous photon correlation spectroscopy and multiscale computer simulations. *J. Phys. Chem. C Nanomater. Interfaces*. **118** (29), 16199–16208. (2014). 25221633 10.1021/jp5034068 PMC4159775

[9] Stetefeld, J., McKenna, S. A. & Patel, T. R. Dynamic light scattering: a practical guide and applications in biomedical sciences. *Biophys. Rev.* **8** (4), 409–427. 10.1007/s12551-016-0218-6 (2016). 28510011 10.1007/s12551-016-0218-6 PMC5425802

[10] Bhirde, A. A., Sindiri, S., Calco, G. N., Aronova, M. A. & Beaucage, S. L. Algorithm-driven high-throughput screening of colloidal nanoparticles under simulated physiological and therapeutic conditions. *Nanoscale* **9** (6), 2291–2300. 10.1039/c6nr08579b (2017). 28127597 10.1039/c6nr08579b

[11] Dahal, E. et al. Structural evaluation of an amyloid fibril model using small-angle x-ray scattering. *Phys. Biol.* **14** (4), 046001. 10.1088/1478-3975/aa776a (2017). 28585521 10.1088/1478-3975/aa776a

[12] Bhirde, A. et al. High performance size exclusion chromatography and High-Throughput dynamic light scattering as orthogonal methods to screen for aggregation and stability of monoclonal antibody drug products. *J. Pharm. Sci.* **109** (11), 3330–3339. 10.1016/j.xphs.2020.08.013 (2020). 32835703 10.1016/j.xphs.2020.08.013

[13] Xie, T. et al. The ELISA detectability and potency of pegfilgrastim decrease in physiological conditions: key roles for aggregation and individual variability. *Sci. Rep.* **10** (1), 2476. 10.1038/s41598-020-59346-z (2020). 32051479 10.1038/s41598-020-59346-z PMC7016140

[14] Bhirde, A. A., Chiang, M. J., Venna, R., Beaucage, S. & Brorson, K. High-Throughput In-Use and stress size stability screening of protein therapeutics using Algorithm-Driven dynamic light scattering. *J. Pharm. Sci.* **107** (8), 2055–2062. 10.1016/j.xphs.2018.04.017 (2018). 29715479 10.1016/j.xphs.2018.04.017

[15] Kim, M. et al. Failure mode identification of insulin drug Products - Impact of relevant stress conditions on the quality of the drug. *J. Pharm. Sci.* **111** (9), 2451–2457. 10.1016/j.xphs.2022.06.013 (2022). 35753411 10.1016/j.xphs.2022.06.013

[16] Hassan, L. A., Al-Ghobashy, M. A. & Abbas, S. S. Evaluation of the pattern and kinetics of degradation of adalimumab using a stability-indicating orthogonal testing protocol. *Biomed. Chromatogr.* **33** (12), e4676. 10.1002/bmc.4676 (2019). 31389037 10.1002/bmc.4676

[17] Hermosilla, J. et al. Comparative stability studies of different Infliximab and biosimilar CT-P13 clinical solutions by combined use of physicochemical analytical techniques and Enzyme-Linked immunosorbent assay (ELISA). *BioDrugs* **33** (2), 193–205. 10.1007/s40259-019-00342-9 (2019). 30875076 10.1007/s40259-019-00342-9

[18] Trabik, Y. A., Moenes, E. M., Al-Ghobashy, M. A., Nebsen, M. & Ayad, M. F. Analytical comparability study of anti-CD20 monoclonal antibodies rituximab and obinutuzumab using a stability-indicating orthogonal testing protocol: effect of structural optimization and glycoengineering. *J. Chromatogr. B Analyt Technol. Biomed. Life Sci.* **1159**, 122359. 10.1016/j.jchromb.2020.122359 (2020). 32920338 10.1016/j.jchromb.2020.122359

[19] Giuliani, A. The application of principal component analysis to drug discovery and biomedical data. *Drug Discov. Today*. **22** (7), 1069–1076. 10.1016/j.drudis.2017.01.005 (2017). 28111329 10.1016/j.drudis.2017.01.005

[20] Shukla, M. K. et al. Identification of monoclonal antibody drug substances using non-destructive Raman spectroscopy. *Spectrochim. Acta Mol. Biomol. Spectrosc.* **299**, 122872. 10.1016/j.saa.2023.122872 (2023). 10.1016/j.saa.2023.122872 37209478

