# Supplementary material for: Innovative approach to support therapeutic proteins’ similarity in hydrodynamic size using high-throughput dynamic light scattering and forced degradation
Source: Sci Rep. 2025 Nov 22;15:41481. doi: 10.1038/s41598-025-97377-6 (PMC12644871; doi:10.1038/s41598-025-97377-6)
Supplement: Supplementary file 1 — Supplementary Material 1 [file 41598_2025_97377_MOESM1_ESM.docx]

**Innovative approach to support therapeutic proteins’ similarity in hydrodynamic size using high-throughput dynamic light scattering and forced degradation**

Ashwinkumar Bhirde,^1*^ Siri Harish,^1^ Nicholas Trunfio,^1^ Isabella F de Luna,^1^ William Smith,^2^ Qiong Fu,^3^

^1^Division of Product Quality Research VI, Office of Pharmaceutical Quality Research, Office of Pharmaceutical Quality, Center for Drug Evaluation and Research, U.S. Food and Drug Administration, 10903 New Hampshire Avenue, Silver Spring, MD, 20993, United States

^2^Division of Product Quality Research V, Office of Pharmaceutical Quality Research, Office of Pharmaceutical Quality, Center for Drug Evaluation and Research, U.S. Food and Drug Administration, 10903 New Hampshire Avenue, Silver Spring, MD, 20993, United States

^3^Division of Product Quality Assessment XIV, Office of Pharmaceutical Quality Assessment III, Office of Pharmaceutical Quality, Center for Drug Evaluation and Research, U.S. Food and Drug Administration, 10903 New Hampshire Avenue, Silver Spring, MD, 20993, United States

**Supporting Figures**


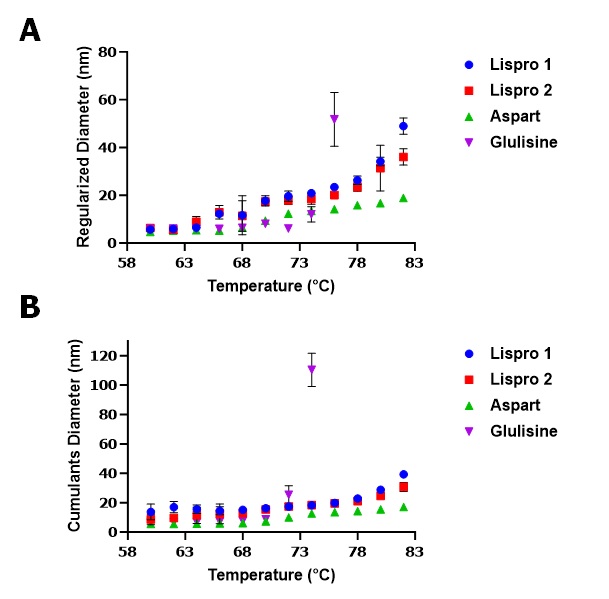


**Figure S1:** Hydrodynamic (D_h_) size changes of insulin drug products (DPs) within the Sweet Spot temperature ramping condition with 2-minute hold time and 3 ^o^C increment temperature. (A) D_h_ sizes (regularization method) of insulin DPs aspart, glulisine, lispro 1 and lispro 2 stressed at 60 – 80 ^o^C range. (B) D_h_ sizes (cumulants method) of insulin DPs aspart, glulisine, lispro 1 and lispro 2 stressed at 60 – 80 ^o^C range. Regularized (A) sweet spot range clearly shows D_h_ size similarity between the insulin lispro DPs lispro 1 lispro 2. In case of glulisine was not a common occurrence but a rare event.

**
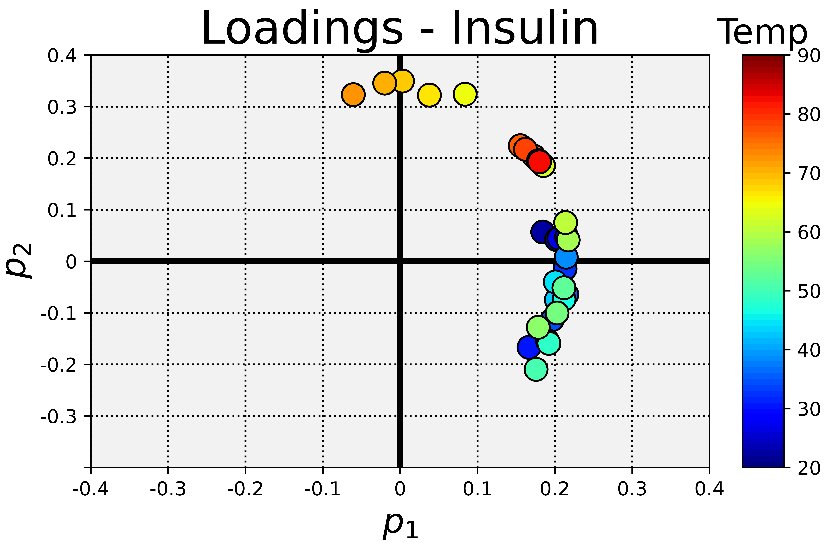

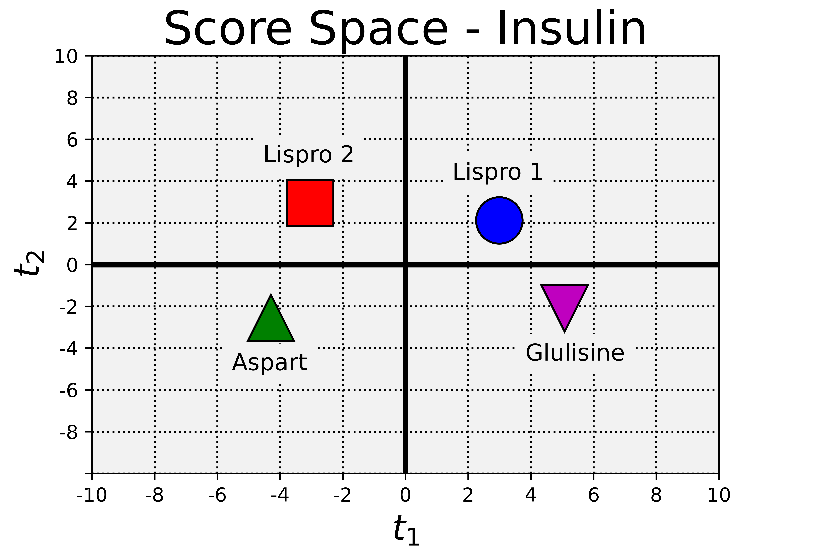
**

**B**

**A**

**Figure S2:** Principal component analysis modeling of the Hydrodynamic (D_h_) size changes of insulin drug products (DPs) within the Sweet Spot temperature ramping condition with 2-minute hold time and 3 ^o^C increment temperature. The score space (A) and loadings (B) for the insulin products. They show that lispro 2 and lispro 1 diameters tend to increase more rapidly as the temperature increased relative to aspart and glulisine.


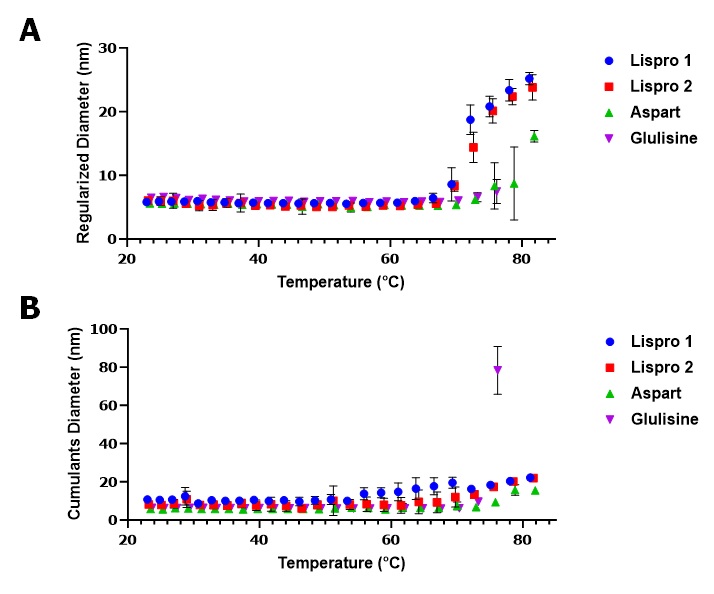


**Figure S3:** Hydrodynamic (D_h_) size changes of insulin drug products (DPs) under continuous temperature ramping condition with no hold time and 3 ^o^C increment temperature. (A) D_h_ sizes (regularization method) of insulin DPs lispro 1, lispro 2, aspart and glulisine stressed at 22 – 82 ^o^C range. (B) D_h_ sizes (cumulants method) of insulin DPs lispro 1, lispro 2, aspart, and glulisine stressed at 22 – 82 ^o^C range. Continuous ramping approach does not provide a clear D_h_ size change pattern between the insulin DPs tested.


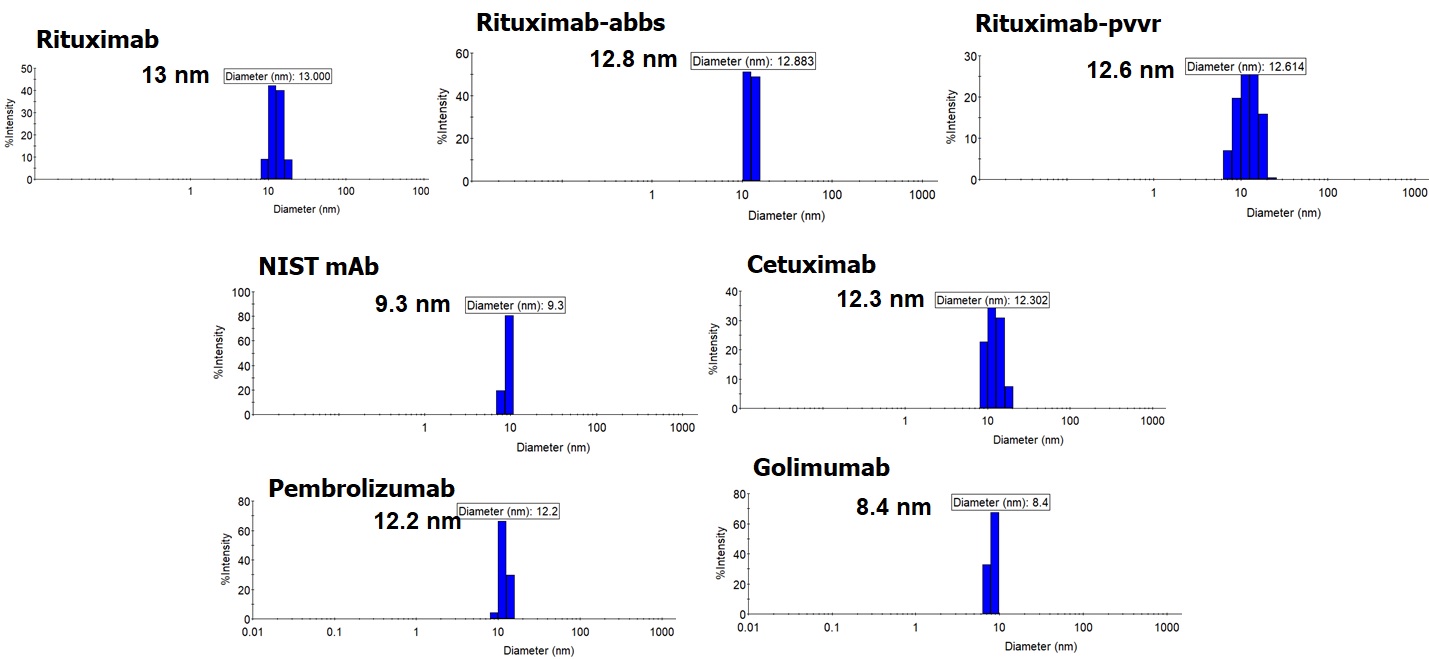


**Figure S4:** Signature hydrodynamic (D_h_) size peaks of mAb DPs in solution at room temperature. All mAbs show a signature peak between 8-13 nm.


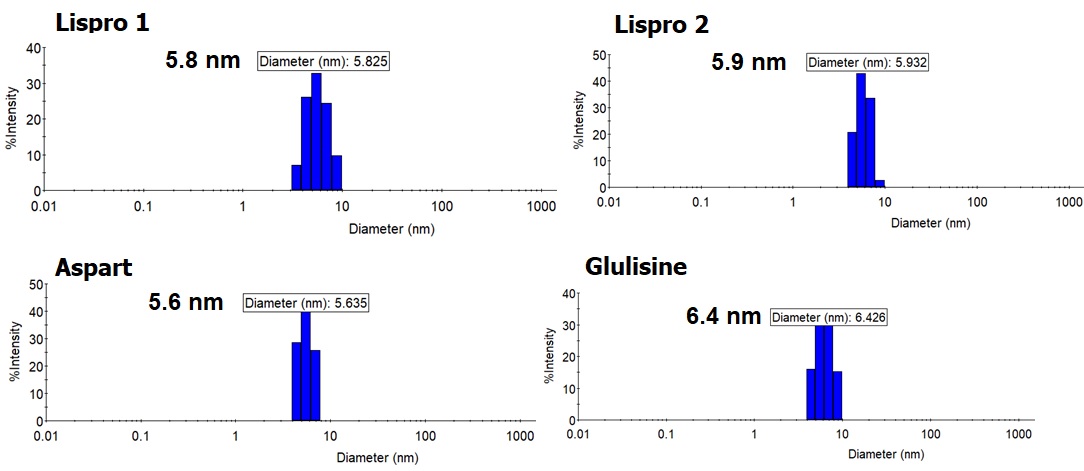


**Figure S5:** Signature hydrodynamic (D_h_) size histograms of insulin DPs in solution at room temperature. All insulin DPs tested show a signature peak between 5-7 nm.


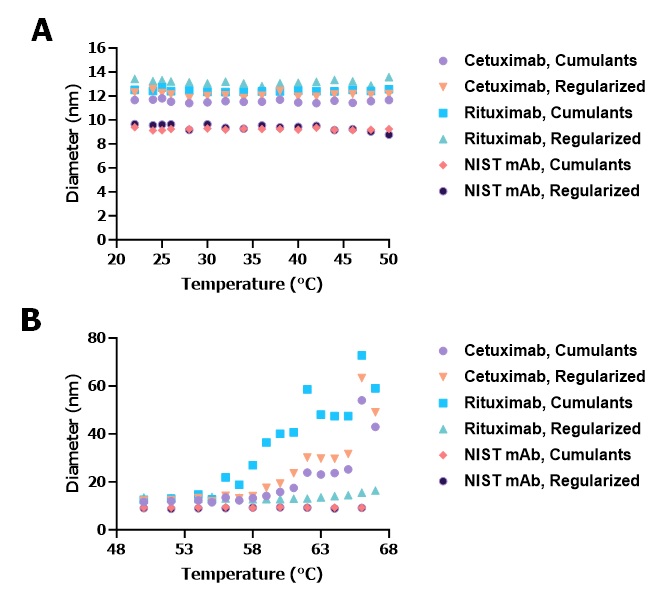


**Figure S6:** Cumulants and regularization hydrodynamic (D_h_) size changes of monoclonal antibody (mAb) drug products (DPs) within the no change and sweet spot temperature ramping condition with 2-minute hold time and 3 ^o^C increment temperature. (A) D_h_ sizes (cumulant and regularization method) of mAb DPs NIST mAb, cetuximab, and rituximab stressed at 22 – 50 ^o^C range. (B) D_h_ sizes (cumulant and regularization method) of mAb DPs NIST mAb, cetuximab, and rituximab stressed at 50 – 67 ^o^C range. Both cumulants and regularization showed similar D_h_ size change trends for the no-change range (A). Regularized (B) showed a D_h_ size change pattern for the sweet spot range. However, it was challenging to observe a D_h_ size change pattern for the sweet spot range using cumulants.

|  | **Sweet Spot method to support analytical similarity of therapeutic proteins using DLS** | |
| --- | --- | --- |
| 1 | Signature peak | Establishing signature peak for candidate mAb or insulin drug product with the respective approved drug product using relevant controls. |
| 2 | Temperature range | All mAbs and Insulins show same size similarity within a certain temperature range. To identity differences and similarities, temperature range needs to be determined. Below a certain temperature, no change in size between the products and the controls tested can be observed. Beyond a certain temperature, all products tested will behave differently. |
| 3 | Temperature increment | Having a wider or too smaller incrementing temperature may not adequately provide the necessary data points for similarity therefore temperature increment should be optimized. |
| 4 | Hold time | Having an adequate hold time is a must to obtain meaningful similarity data. |

**Table S1:** Four key steps in establishing hydrodynamic size similarity of therapeutic protein drug products using the Sweet Spot method. Once established, the data needs to be evaluated for repeatability.
